# Evolving challenges and strategies for fungal control in the food supply chain

**DOI:** 10.1016/j.fbr.2021.01.003

**Published:** 2021-06

**Authors:** Catheryn R. Davies, Franziska Wohlgemuth, Taran Young, Joseph Violet, Matthew Dickinson, Jan-Willem Sanders, Cindy Vallieres, Simon V. Avery

**Affiliations:** a School of Life Sciences, University of Nottingham, University Park Campus, Nottingham, United Kingdom; b School of Biosciences, University of Nottingham, Sutton Bonington Campus, Loughborough, United Kingdom; c Unilever Foods Innovation Centre, Bronland 14, 6708 WH Wageningen, the Netherlands

**Keywords:** Agrichemicals, Antimicrobial resistance, Food spoilage, Phytopathogens, Spoilage fungi

## Abstract

Fungi that spoil foods or infect crops can have major socioeconomic impacts, posing threats to food security. The strategies needed to manage these fungi are evolving, given the growing incidence of fungicide resistance, tightening regulations of chemicals use and market trends imposing new food-preservation challenges. For example, alternative methods for crop protection such as RNA-based fungicides, biocontrol, or stimulation of natural plant defences may lessen concerns like environmental toxicity of chemical fungicides. There is renewed focus on natural product preservatives and fungicides, which can bypass regulations for ‘clean label’ food products. These require investment to find effective, safe activities within complex mixtures such as plant extracts. Alternatively, physical measures may be one key for fungal control, such as polymer materials which passively resist attachment and colonization by fungi. Reducing or replacing traditional chlorine treatments (*e.g.* of post-harvest produce) is desirable to limit formation of disinfection by-products. In addition, the current growth in lower sugar food products can alter metabolic routing of carbon utilization in spoilage yeasts, with implications for efficacy of food preservatives acting via metabolism. The use of preservative or fungicide combinations, while involving more than one chemical, can reduce total chemicals usage where these act synergistically. Such approaches might also help target different subpopulations within heteroresistant fungal populations. These approaches are discussed in the context of current challenges for food preservation, focussing on pre-harvest fungal control, fresh produce and stored food preservation. Several strategies show growing potential for mitigating or reversing the risks posed by fungi in the food supply chain.

## 1 Introduction

The global human population is predicted to rise to 9.7 billion by 2050 (United Nations, 2019). To contend with this, global production of edible crops may need to increase by up to 119 % (Berners-Lee *et al.*, 2018). Alternatively, waste and spoilage of foods must be dramatically reduced. Therefore, the food industry faces serious challenges to meet current and projected demand. Besides issues relating to food transport and storage infrastructure, water resilience, and the impacts of climate change, there is too much food waste including through livestock and crop disease. Fungi (moulds and yeasts) are major contributors to this, undermining resilience of the food supply chain at key stages from the infection of seeds and growing crops to spoilage post-harvest and in processed foods, during processing, transport and storage. These concerns are despite the positive contributions of other fungi to food production, *e.g.* organic acids, mycoprotein, baking, brewing, cheese production.

Phytopathogenic fungi are responsible for up to 20 % loss of global crop yield, enough food to feed up to 600 million people annually (Anonymous, 2017). Fungal diseases of the five most cultivated food crops worldwide were estimated to destroy at least 125 million tonnes of produce every year (Fisher *et al.*, 2012; Savary *et al.*, 2012). Some fungi, such as the rice pathogen *Magnaporthe oryzae*, can cause yield losses of up to 100 % (Musiime *et al.*, 2005). In addition, animal feed contaminated with fungi can give rise to 5–10 % reductions in milk production from cattle due to the formation of mycotoxins (Simion, 2018), and livestock themselves are susceptible to fungal infections (Ahmad and Gholib, 2016). Phytopathogenic fungi cost the global economy several hundred billion USD each year and untold misery for the farming sector (Birren *et al.*, 2002).

Fungi also cause serious problems at subsequent stages of food production. An additional 10 % of crops are destroyed post-harvest (Fisher *et al.*, 2018). Furthermore, spoilage fungi are a major contributor to the ruination of perishable foods including cheese, carbonated drinks and condiments. Filamentous fungi such as *Aspergillus niger* are a threat to solid, fresh produce such as fruit, while spoilage yeasts are capable of spoiling liquid preserves and beverages including those formulated to counter colonisation by spoilage microorganisms (Pitt and Hocking, 2009). Whereas use of preservatives and factory hygiene (*e.g.* aseptic process lines) mitigates against spoilage by many fungal species, certain spoilage yeasts are characterised by an extreme resistance to preservatives and can persist in storage environments inhospitable to unspecialised microorganisms (Davenport, 1998). Some spoilage fungi also have the potential to endanger lives through the production of mycotoxins, and the opportunistic infection of immunocompromised hosts (Benedict *et al.*, 2016; WHO, 2018).

In addition to the above, there are several emerging fungal threats to the food supply chain. These include emerging pathogens, spoilage organisms and an increased prevalence of fungicide/preservative resistant strains; exacerbated by use of crop monocultures, over-use of single-target fungicides and changing food formulations to meet evolving market demands. Other wider issues such as climate change, enabling fungal phytopathogens to spread poleward into warming climates, also contribute and pose different growing threats for different countries (Avery *et al.*, 2019; Bebber *et al.*, 2013).

In this focused review, we highlight key current issues presented by fungi for food production and supply, and strategies for tackling these emerging pressures on food supply chains. We start from the point of the food sources and work forward to consumption (fresh produce and stored foods).

## 2 Pre-harvest fungal control

### 2.1 Evolving challenges

Controlling fungal pathogens of crops is a cornerstone for food security. With pre-harvest losses to fungi still exceeding 20 % of total yield (Fisher *et al.*, 2018), it is not surprising that the global fungicides market is valued at $13.4 billion per annum (Garside, 2019). Resistance to the currently available fungicides is a threat to crop protection. Fungicide resistance has emerged in many of the most prevalent fungal phytopathogens (Savary *et al.*, 2012). Spread of resistance is accelerated by factors such as the airborne dissemination of fungal spores (Torriani *et al.*, 2009). Indiscriminate use of fungicides has caused health and environmental problems associated with chemical residues and the selection of resistant pathogens, leading to the introduction of more restrictive regulations on fungicide use. In addition, the costs and time-frame for developing and commercialising new fungicides are sufficiently high that it is usually only major crops that may attract the necessary agrichemical-industry investment (McDougall, 2016).

To help counter these problems, one focus has been on resistance breeding of crop cultivars that can resist fungal infection (Ellis *et al.*, 2014). An important example has been the discovery and subsequent cultivation of recessive alleles of the *Mlo* gene within the economically valuable cereal barley. The mutant *mlo* alleles can provide durable, broad spectrum resistance to powdery mildew caused by the *Blumeria graminis* fungus. The allele *mlo-*11 has been widely exploited in barley cultivars across Europe since its discovery several decades ago (Ge *et al.*, 2016; Jørgensen, 1992). Another example of successful resistance breeding is observed in the control of Fusarium wilt of banana caused by *Fusarium oxysporum*. Here, genetic resistance was the main control strategy until the 1990s when a strain (*F. oxysporum* f. sp. *cubense* tropical race 4, *Foc* TR4) virulent to the resistant cultivar emerged (Bubici *et al.*, 2019). Thus, although there have been notable successes in the development of resistance cultivars, this is clouded by several challenges (Li *et al.*, 2020). Besides emergence of new fungal strains, a further drawback of resistant cultivars is that elevated resistance often negatively influences plant development, which is a mounting concern as improvements to plant yields are needed to meet expected future demand (Ning *et al.*, 2017). Concerning the use of GM approaches to achieve resistance, studies show that consumers would prefer to buy non-GM foods over GM foods (Lefebvre *et al.*, 2018) and therefore careful, educational marketing is needed to promote widespread acceptance and implementation of these.

**Fig 1:**
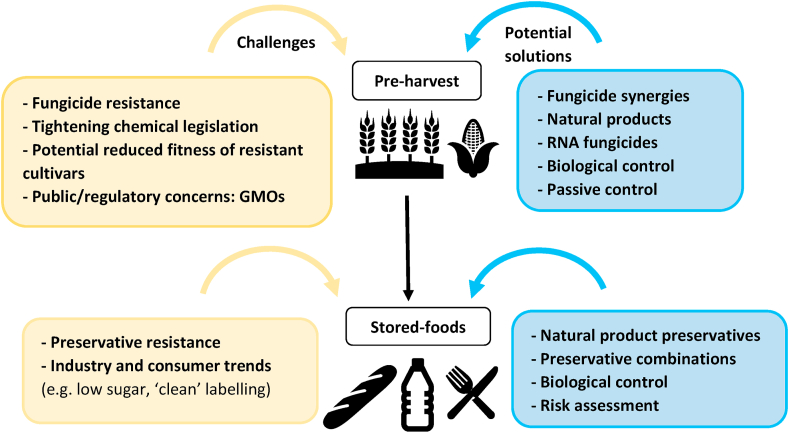
Summary of challenges and potential solutions in pre-harvest control and stored food preservation from fungi. Yellow boxes indicate challenges. Blue boxes indicate examples of potential control strategies discussed in this review. GMO, genetically modified organism.

We summarize below a non-exhaustive list of strategies that are being developed or considered as alternatives to traditional methods of pre-harvest fungal control (see also Fig 1). For additional coverage of challenges for fungal phytopathogen control that are linked to climate change, agronomical practices and anthropogenic factors (*e.g.* crop trade, transport and pathogen introduction), the reader is referred to Fones *et al.* (2020).

### 2.2 Potential control strategies

#### 2.2.1 Fungicide synergies

Application of fungicide mixtures to crops is recommended practice (Brent and Hollomon, 2007). For example, mixtures from Bayer and Syngenta that are currently on the market generally contain combinations of two fungicides with distinct modes of action and which have additive effects on fungal inhibition. Mixtures where the fungicides may act synergistically have added appeal as they allow use of decreased doses of each compound for a desired level of inhibition, potentially lowering costs and environmental impact. Given that the use of chemical fungicides remains an important component of available crop protection strategies (Keulemans *et al.*, 2019), the exploitation of fungicide synergies helps address the demand for reduced chemicals usage while retaining the advantages of potent fungal protection. However, this strategy must be used carefully as resistance developed to one compound will abrogate the effect of the combination, *i.e.*, the second agent may be at too low a concentration to inhibit fungal growth by itself. These risks may be offset by the incorporation of such mixtures into rotational fungicide programmes. Combinations of agents acting synergistically against fungal phytopathogens have been reported in the literature (Moreno-Martinez *et al.*, 2015; Vallieres *et al.*, 2018) but this approach is as yet poorly reported for field applications.

#### 2.2.2 Natural product inhibitors

Sources of natural products (NPs) present opportunities for the discovery of natural fungal inhibitors (some advantages of NPs are addressed later in this article). NPs with an inherent fungicidal activity have been isolated from diverse sources, ranging from plants and animals to marine organisms (Dong *et al.*, 2020). Promising NPs can be further improved by chemical optimisation (although derivatives may require evaluation and registration). Such optimisation was described recently with the example of luotonin A, a plant-extracted quinoline alkaloid with existing inhibitory activity against multiple fungal phytopathogens, where several synthesised NP-analogues displayed activity comparable to that of the widely used fungicide azoxystrobin (Yang *et al.*, 2020). A further example of enhancing NP potential is evident from a growing number of available biofumigant treatments. Biofumigation describes the incorporation of freshly harvested cover crop into soil, which allows crop breakdown to release glucosinolate products (*i.e.* isothiocyanates) that act to ‘sanitise’ the soil. This process also upholds potential fungal inhibitory properties (Calmes *et al.*, 2015). In applying biofumigation for control of the wheat pathogen *Fusarium graminearum*, mulch layer treatments composed of cover crops (*e.g.* white mustard, Indian mustard, berseem clover) have been shown to suppress infection, decrease the mycotoxin burden and improve grain yield (Drakopoulos *et al.*, 2020).

#### 2.2.3 RNA-based “fungicides”

Fungi-specific control strategies help to address environmental concerns with fungicides and potential toxicity through the food chain. RNA interference (RNAi) allows the expression of double-stranded RNA molecules (dsRNAs) or small RNAs (sRNAs) that can specifically target virulence-related genes of fungal phytopathogens to help protect plants from fungal infections (Cai *et al.*, 2018, 2019; Huang *et al.*, 2019). The inhibitory RNAs can be delivered either by transgenic expression of dsRNAs, though that approach carries GMO-related concerns, or direct application of dsRNAs or sRNAs into host plants to establish targeted silencing. Spray application of RNAs targeting critical fungal genes has been shown to inhibit infections by phytopathogens such as *F. graminearum*, *Botrytis cinerea* and *Sclerotina sclerotiorum* on diverse crops (McLoughlin *et al.*, 2018; Wang *et al.*, 2016). Evidence suggests that RNAs can be either directly taken up by the pathogens, or can accumulate in the plant from where the RNAs are transferred into fungal cells (Wang and Jin, 2017).

#### 2.2.4 Biocontrol and stimulation of natural plant defences

Biocontrol has also been studied as a more natural alternative to chemical fungicides for combatting diverse fungal infections in agriculture (Bubici *et al.*, 2019; Chen *et al.*, 2018; Vos *et al.*, 2015). In occupying similar environmental niches, several species of the fungal genus *Trichoderma* can limit the proliferation of major pathogens such as *Fusarium* spp. (Bubici *et al.*, 2019) and *B. cinerea* (Vos *et al.*, 2015). Biocontrol involves competition for space and nutrients, synthesis of antifungal substances and secondary metabolites (Rashad and Moussa, 2020) or biological triggering of plant resistance (Harman *et al.*, 2004; Hermosa *et al.*, 2013). Currently, the use of biocontrol agents does not provide complete control and, therefore, they are commonly deployed in combination with fungicides (Hua *et al.*, 2018). In another form of control, plant signalling molecules such as salicylic acid and jasmonic acid can induce resistance of plants to fungal pathogens (Park *et al.*, 2007; Robert-Seilaniantz *et al.*, 2011) opening possibilities for exploitation in the management of fungal diseases (Liu *et al.*, 2019). Relevant also to post-harvest fungal control (discussed later), UV-C treatment applied periodically in low doses induces a response of the plant that includes increased production of secondary metabolites and increased resistance to fungal pathogens such as *B. cinerea* (Jin *et al.*, 2017; Scott *et al.*, 2019).

#### 2.2.5 Passive approaches for phytopathogen control

Resistance to chemical fungicides is already well reported and several of the above strategies could also be undermined by emergence of resistance, as they impose a selective pressure on the fungi to adapt (or be inhibited/killed). Recently, a small group of (meth-)acrylate polymers were characterised which effectively block the attachment of *B. cinerea* spores to spray-coated leaf surfaces (Vallieres *et al.*, 2020). Similar materials also resisted attachment by *Zymoseptoria tritici*. This anti-attachment effect of the polymers is passive, impairing the first step of infection without killing the pathogens (or damaging the leaves). Consequently, this strategy could exert less selective pressure for resistance than fungicides, reinforced by the fact that development of resistance should require a gain in the ability to attach, potentially raising greater evolutionary hurdles (Cohen-Gihon *et al.*, 2011). Chemically-related polymers have already been used as adjuvants in commercial fungicide formulations, *e.g.* to facilitate active-fungicide delivery, indicating safe use of these types of material in the field. By reducing the need for active chemicals, passive approaches for combatting fungal infection could offer more sustainable strategies into the future.

## 3 Fungal control in fresh produce

### 3.1 Evolving challenges

Food losses due to fungal spoilage are common for perishable foods such as fruits and vegetables, with sources of contamination ranging from pre-harvest (irrigation water, soil, contaminated seeds) to post-harvest steps (handling, storage and transportation, cross-contamination). Warm and humid climates or extreme weather events further increase the risk of spoilage (Bhilwadikar *et al.*, 2019; FAO, 2019). Fresh produce can be washed with chemical sanitisers to reduce the fungal load. The most common choice for many years has been chlorine treatment in the form of washes with dissolved hypochlorite salts; the application and efficacy of which have been reviewed (Bhilwadikar *et al.*, 2019; Kaczmarek *et al.*, 2019). Alternative chemical treatments include chlorine dioxide (ClO_2_), alcohols, copper sulphate (CuSO_4_), organic acids, hydrogen peroxide (H_2_O_2_), ozone gas (O_3_) and peracetic acid (PAA). A common challenge with most chemical treatments is their effectiveness at dosages and treatment times that do not also adversely affect the produce. Additional concerns with specific treatments are low chemical stability (H_2_O_2_, PAA, O_3_), safety concerns in factories (ClO_2_, PAA, O_3_) and toxicity of residues (CuSO_4_, ClO_2_) (Bastarrachea *et al.*, 2019; Deng *et al.*, 2019); these concerns contribute to the ongoing use of hypochlorite salts. However, when free available chlorine (FAC; pH-dependent mix of ^−^OCl, HOCl and Cl_2_) reacts with organic matter, disinfection by-products (DBPs) can form (Gil *et al.*, 2019; How *et al.*, 2018; Tudela *et al.*, 2019). This represents a health risk both in the processing water and on the washed produce due to the genotoxicity and carcinogenicity of some DBPs (Villanueva *et al.*, 2015). Progress towards more stringent regulations of DBP levels in water (EU, 2018) and demands for clearer food labelling (Gracia and de-Magistris, 2016) underline the importance of controlling DPB formation during food sanitisation. Removal of chlorine and DBPs in the effluent water can be achieved using dechlorination chemicals or activated carbon filters (Gil *et al.*, 2019; Hermant and Basu, 2013). However, these measures add a processing step, and do not help with residues already deposited on the washed produce. Alternative fresh-produce treatments which help address several of the above disadvantages are discussed below. It is noted that certain approaches used in pre-harvest fungal control described in other sections of this review can also have effect post-harvest, including biocontrol agents and natural compounds with antifungal activity (Lamenew *et al.*, 2019; Talibi *et al.*, 2014). Modified atmosphere packaging after harvest, processing and potential sanitising treatments can further increase the shelf life of fresh produce (Mahajan *et al.*, 2014). Different packaging technologies can influence factors such as CO_2_ levels or moisture either passively or actively. For instance, the incorporation of oxygen scavenging material into packaging helped reduce the extent of fungal decay on berries (Niazmand and Yeganehzad, 2020). Choosing the right sanitisation and preservation method(s) will also depend on the application parameters, such as the produce type or the organic load during the sanitisation treatment (Van Haute *et al.*, 2015).

### 3.2 Potential control strategies

#### 3.2.1 Electrolysed water

**Fig 2:**
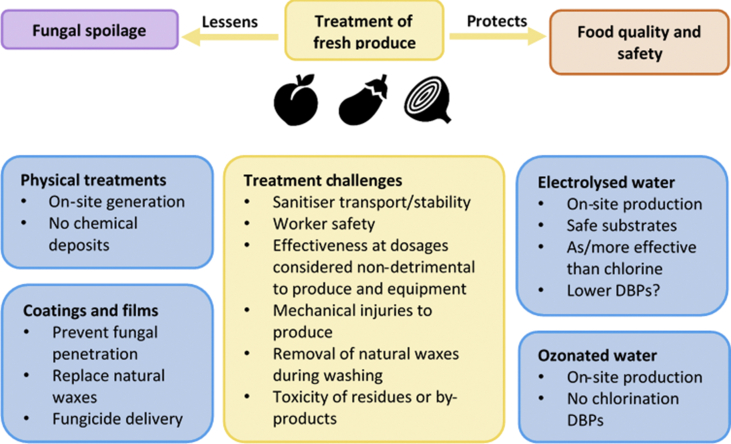
Challenges and potential solutions for post-harvest treatments to control fungal spoilage. Blue boxes, potential solutions and their advantages.

In conventional chlorine treatment of post-harvest produce, calcium hypochlorite is shipped in dry form and needs to be fully dissolved in water to limit damage to the produce, whereas sodium hypochlorite is only stable in solution making shipping more expensive (Mishra *et al.*, 2018). Using electrochemistry, solutions of the required FAC can instead be produced on-site and in response to demand, from cheap, safe ingredients (water and NaCl); the product is termed electrolysed water (EW) (Zhang *et al.*, 2018). The electrolysis of the salt solution yields FAC species. This eliminates the need for transport and handling of concentrated solutions and concerns over long-term stability (Fig 2) (Gil *et al.*, 2015). To prevent chlorine gas formation at low pH, a more neutral or slightly-acidic EW is preferred to acidic EW (Ali *et al.*, 2018). EW has been in use for a number of years, but its potential for treating fresh produce is still being explored and developed (Youssef and Hussien, 2020). The resilience of EW activity to the presence of incidental soil, often associated with fresh produce, has recently been established (Wohlgemuth *et al.*, 2020). When EW is produced, other active species besides FAC are generated, including O_3_, ClO_2_, H_2_O_2_, superoxide (O_2_^−^) and hydroxyl radicals (•OH) (Jeong *et al.*, 2009; Zhang *et al.*, 2018). These additional species may help attain fungal killing at lower FAC concentrations, reducing the formation of chlorinated DBPs during the sanitisation process. Accordingly, viable yeast populations on apple slices were reduced ~10-fold more strongly by EW compared to NaOCl (Graça *et al.*, 2020). EW is reported to have greater effectiveness than NaOCl also against filamentous fungi (Koide *et al.*, 2009). Several companies currently offer commercial EW generating systems that are recommended for fresh produce washing or decontamination of food handling surfaces, for use by the food industry or at home, *e.g.*, EcoloxTech, Taeyoung E&T, Ozo Innovations.

#### 3.2.2 Ozonated water

Although EW might allow the use of lower FAC concentrations (see preceding section), DPBs remain a potential concern (Clayton *et al.*, 2019; Gil *et al.*, 2019; Wang *et al.*, 2019), underlining the desirability for FAC-free alternatives. Ozonated water can be produced by electrolysis using water alone (Lutz *et al.*, 2017; Sekido and Kitaori, 2008). Therefore, it shares with EW the benefits of on-site production from safe substrates, but not the disadvantage of FAC content (Fig 2). Instead, ozone is formed that first decomposes to other reactive species, *e.g.*, hydroxyl radicals (Baggio *et al.*, 2020; Wang *et al.*, 2020) and finally to oxygen and water. Therefore, ozonated water treatment does not usually leave residues on the produce (Brodowska *et al.*, 2018), although certain by-products such as carbonyls or bromates might form in the presence of organic matter or bromide ions (Papageorgiou *et al.*, 2017; Wu *et al.*, 2019). Cravero *et al.* (2018) reported similar levels of yeast killing on grapes treated with EW or ozonated water, at only 5 ppm ozone *versus* 400 ppm FAC in the EW. However, on various fresh-food surfaces, ozonated water (0.7–5 ppm ozone) showed weaker inactivation of fungi than EW (5–34 ppm FAC) (Degala *et al.*, 2020; Koseki and Isobe, 2007), possibly reflecting the difficulty of maintaining sufficient ozone concentrations due to its low stability (Deng *et al.*, 2019). Ozone in aqueous and gaseous phases has been approved by the FDA for use in food for human consumption. Ozonated water generators are commercially available (*e.g.* Absolute Ozone, GreenTeck Global) and several food brands use ozone treatments (Hinkle, 2017). Challenges of ozone treatment include a short half-life (seconds to hours, depending on parameters like water quality), health concerns associated with ozone gas formation and risk of corrosion depending on the ozone concentration (Baggio *et al.*, 2020; Brodowska *et al.*, 2018).

#### 3.2.3 Physical treatments

Physical treatments avoid chemical deposits on the produce (Fig 2). In addition to inactivating microorganisms, UV light may stimulate plant defence systems in fresh fruits and vegetables (Zhang and Jiang, 2019). One promising technology is pulsed light (PL), consisting of broad spectrum electromagnetic radiation (from the UV to the infrared range). It is more effective in these applications than continuous UV treatments, and commercial PL systems are available (Deng *et al.*, 2019). Fungal spoilage of strawberries after 8 d of storage was reduced by 16–42 % after PL treatment for 2–40 s (Duarte-Molina *et al.*, 2016). Ionising irradiation is more common for killing of microflora on dry food products but has gained interest for fresh produce more recently (both for direct killing of pathogens and stimulation of plant defence), although negative effects on quality and critical consumer perception are ongoing challenges (Demirci *et al.*, 2020; Jeong and Jeong, 2018). An additional concern can be regulatory, especially in the EU where some countries limit the use of irradiation to dried herbs, spices and vegetable seasonings whereas the application extends to fresh produce in the US and other parts of the world (Eustice, 2018). Irradiation of quince fruit (1.2–2.1 kGy) reduced spoilage by yeasts and moulds after 5–15 d by up to 3.8–4.9 log (Hussain *et al.*, 2019). On cucumbers, irradiation (2–3 kGy) delayed fungal spoilage (by one week) but finally resulted in higher fungal spoilage (after 28 d), potentially due to softening of the cucumber tissue (Khalili *et al.*, 2017). Atmospheric cold plasma may find applications for in-package sanitation of fresh produce (Deng *et al.*, 2019). Plasma treatments reduced fungal contamination on nuts although long treatment times (>10 min) were necessary to achieve one log reduction (Devi *et al.*, 2017). Better fungal reduction (~3 log) was achieved with in-package plasma treatments (5 min) of strawberries (Misra *et al.*, 2014). Ultrasonic treatments can be combined with chemical treatments (*e.g.* chlorine based) to increase the sanitising efficacy (Demirci *et al.*, 2020). Accordingly, ultrasound alone gave low reductions of yeasts and moulds on green asparagus (~0.3 log) but combination with acetic acid + gibberellic acid increased the effect to ~1.3 log (acids alone: ~0.6 log) (Wang and Fan, 2019). Other potential methods are high hydrostatic pressure or pulsed electric fields but so far they are reported mostly for processed produce such as fruit juices (Demirci *et al.*, 2020; Pinto *et al.*, 2020). Combining a heat treatment with high pressure markedly improved inactivation of fungal ascospores in a strawberry puree (Timmermans *et al.*, 2020). Physical treatments (heat, irradiation, PL, plasma) are also commonly applied to sanitise food packaging, reducing the risk of spoilage during storage and transport (Demirci *et al.*, 2020). In comparison to chemical treatments, potential drawbacks of physical methods are that they do not remove dirt and plant debris and that they may lead to physical (*e.g.* thermal) damage to the produce. Additionally, the described methods may be challenging to scale up and implement in high-throughput industrial settings (Ali *et al.*, 2018; Demirci *et al.*, 2020; Deng *et al.*, 2019).

#### 3.2.4 Coatings/films

Coatings and films are commonly used in citrus packing houses and help replace the natural waxes that can be lost during washing and handling of the fruit. In general, films are prepared by a casting process before application onto the surface of the food product, whereas coatings involve the formation of the protective layer directly on the surface. Coatings and films have similar compositions; they can be made of hydrocolloids such as proteins and polysaccharides, lipids like waxes, acylglycerols or fatty acids, and resins (Palou *et al.*, 2015). These can help protect against physical damage (which usually accelerates fungal decay) and present a matrix for delivering synthetic or natural-product food preservatives, or biocontrol agents, which are released gradually to protect against fungal colonization (Palou *et al.*, 2015). Chitosan and chitosan-based coatings are attractive as chitosan is a natural biopolymer with fungicidal properties and has properties suited to forming films and coatings. Beneficial effects of chitosan coatings for controlling post-harvest fungal infections have been reported across diverse horticultural products, including citrus (Chaudhary *et al.*, 2020).

## 4 Preservation of stored foods from fungal spoilage

### 4.1 Evolving challenges

At the consumer level in industrialised countries, spoilage and concern over spoilage are factors contributing to losses of up to 30 % of stored-foods (FAO, 2011). Spoilage is associated with negative effects on the organoleptic quality of products, *e.g.*, aesthetic, texture, taste properties. Fungi are major food-spoilage organisms, including both yeasts and moulds. Their disruptive ability is heightened by their collective ability to colonise diverse, harsh environments (Selbmann, 2019).

The airborne dispersal of ubiquitous fungal spores (sexual or asexual) means that foods can readily become contaminated, colonised and consequently spoiled. This can be initiated during processing, storage or handling by the manufacturer, retailer or the consumer, culminating in loss of the food and, potentially, costly product recalls (Leyva Salas *et al.*, 2017). Furthermore, as mentioned above, certain fungi associated with foods produce mycotoxins as secondary metabolites, which represent a danger to health, including carcinogenicity (Krisch *et al.*, 2011). This is compounded by the fact that mycotoxins can be very stable, exhibiting a degree of temperature- and acid-tolerance (Filtenborg *et al.*, 1996). Consequently, control or monitoring of mycotoxins in foods is challenging and demands careful management.

At neutral pH and high water-activity, the most abundant spoilage microorganisms are bacteria. However, while some bacterial species are adapted to either low pH or high osmolarity conditions, the combination of these factors in many preserved foods creates environments more favourable to yeasts and filamentous fungi. To prevent fungal spoilage, these conditions combined with low temperature storage and use of preservatives like weak organic acids can be effective. However, certain yeasts can overcome these measures. Growth of *Rhodotorula mucilaginosa* is relatively resistant to refrigeration and typically spoils refrigerated dairy products while *Candida parapsilosis* is resistant to salt stress and often associated with spoilage of preserved meats (Pitt and Hocking, 2009; Stratford *et al.*, 2019). Another example is the yeast *Zygosaccharomyces bailii* which is extremely resistant to weak acids (Stratford *et al.*, 2013), and typically spoils foods such as soft drinks and condiments (Martorell *et al.*, 2007). This apparent specialisation allows spoilage yeasts to colonise certain food environments that are inhospitable to most moulds and bacteria, and which cannot be made harsher within acceptable parameters of taste or product safety (Anonymous, 1995).

The sugar levy on soft drinks introduced by the UK government in 2018 and similar moves made by certain other governments has brought new preservation challenges to the fore, as migration to lower-sugar products may influence the metabolism and preservative-resistances of fungal spoilage species. Preservatives like sorbic acid target fungal respiration (Stratford *et al.*, 2020), and spoilage yeasts have a greater relative reliance on respiration (*versus* fermentation) for growth when in lower-sugar drinks. Accordingly, such preservatives are likely to exert improved control of yeast spoilage in low sugar products. However, other formulation changes to help accommodate lower sugar content in certain products, such as raised pH, may promote new spoilage problems. It has been reported that sugar-substituted foods such as cake-type bakery products can be more prone to fungal spoilage (Rodriguez *et al.*, 2016). The role that high levels of sugar can have in preserving shelf life may also need to be considered when substituting sugar with substances without such properties, and additional preservation methods could need to be employed. Modification of product formulations to meet new regulations must be done with care to minimise the risks of fungal spoilage.

Other challenges currently faced by the food industry include the growing demand from consumers for the avoidance of plastic packaging and the appropriate use of ‘green’ and safe preservatives, *e.g.* clean-label products or approved E-numbers. This has accelerated research efforts to find effective solutions such as natural-product preservatives to lower the use of synthetic chemicals or other additives that require labelling and which raise consumer concerns (Campêlo *et al.*, 2019). Potential strategies for tackling some of the different challenges are addressed below and are summarised in Fig 1.

### 4.2 Potential control strategies

#### 4.2.1 Natural products and biopreservation

In the preservation of stored foods from spoilage, the use of NPs presents an emerging alternative to current control measures. NPs that are natural constituents of the product itself or added NPs that may provide a texture or flavouring contribution, for example, do not necessarily require labelling and therefore also satisfy the consumer pressure for ‘clean’ labelling. One area of NP exploitation is in the antifungal properties of certain essential oils (EOs) for the preservation of stored foods (Garcia and Copetti, 2019). European Regulation No. 1334/2008 defines EOs and their active components as flavouring preparations and substances, respectively. Despite this, every use of EOs for human consumption should be taken with caution due to potential toxicological effects (Debonne *et al.*, 2018). One study incorporated EOs from clove and oregano oil-in-water nanoemulsions and demonstrated their fungicidal potential against *Z. bailii* in salad dressings, inhibiting the yeast with MICs of 1.75 mg mL^−1^ (Ribes *et al.*, 2019). While promising, further optimisation can be needed to meet industry standards, *e.g.*, standards for ambient storage of dressings. The complex and intense flavours commonly associated with EOs must be considered and evaluated for any proposed food use (Debonne *et al.*, 2018; Ribes *et al.*, 2019). EOs from citrus peel, for instance, displayed strong growth inhibitory activity but the treatment had undesirable organoleptic impacts on the treated bread (Rehman *et al.*, 2007).

A variety of weak organic acids, widely used in food preservation, were historically isolated from natural sources. These include acetic, propionic, sorbic and benzoic acids. Except for acetic acid, these preservatives are now produced commercially through chemical synthesis from petrochemicals (Lück and Jager, 1997). While propionic and benzoic acids are naturally occurring in a variety of foods (Yun *et al.*, 2019), weak acids produced through chemical synthesis or highly refined from ‘natural’ sources are classed as artificial preservatives, conflicting with current market pressures.

Biopreservation, such as through the use of lactic acid bacteria (LAB) and certain antagonistic yeasts, has attracted much attention because these organisms can exhibit inherent and potent fungal-inhibitory properties. Certain antifungal products from LABs, including organic acids, bacteriocins and fatty acids that disrupt the fungal cell membrane, are produced by species of genera such as *Lactobacillus* and *Streptococcus* and have gained FDA and EU approval for use as food preservatives (Oliveira *et al.*, 2014; Ribes *et al.*, 2018). These have been used to extend the shelf-life of refrigerated dairy products such as cheese (by at least 3 weeks) and yoghurt (Leyva Salas *et al.*, 2018; Schwenninger and Meile, 2004). Antagonistic yeasts inhibit growth of other fungi through proposed mechanisms including competition for nutrients and space or synthesis of antifungal hydrolases (El Ghaouth *et al.*, 2003; Ribes *et al.*, 2018). Moreover, this inhibitory activity is achieved without toxic metabolite by-production. Despite this only a few yeasts are currently approved for use as biocontrol products. Antagonistic yeasts include certain *Cryptococcus* and *Candida* species, among other genera. These can be introduced to foods by techniques such as spraying onto fruits to control post-harvest fungal spoilage, by *B. cinerea* for example (Wei *et al.*, 2014).

#### 4.2.2 Preservative combinations

Preservative resistance and heteroresistance (variation between individual cells within a population) is a major concern for food quality and safety. Some organisms commonly associated with food spoilage are inherently more resistant to weak acid preservatives, for example *Z. bailii* (Stratford *et al.*, 2013). Furthermore, rare cells or spores can be hyper-resistant to the thresholds of food preservatives allowed in different products, such as soft drinks (Geoghegan *et al.*, 2020; Stratford *et al.*, 2014). One approach to combatting this particular problem could be the use of preservative combinations, where each agent has a different mode of action so lowering the chances of rare-cell resistance (to both agents). This of course would be less desirable where it raises the chemical load of the food and/or the complexity of food production due to additional ingredients and labelling. The chemical load from combinations is lowered where these may have a synergistic interaction, *i.e.*, where the combination effect is greater than anticipated from a simple sum of the individual effects, so enabling reduced dosages for equivalent effect. This could also reduce the likelihood of effects on taste by NPs, for example. However, unlike other applications (*e.g.*, therapeutic drugs), to date there are few published research papers on synergistic preservative combinations tackling fungal spoilage. Synergistic growth inhibition of the spoilage yeast *Z. bailii* was achieved with different chemical combinations comprising a sulphate-transport inhibitor and an aminoglycoside antibiotic (Moreno-Martinez *et al.*, 2015). In this case, regulatory constraints would restrict the use of antibiotics to control *Z. bailii* in products intended for human consumption, but such studies suggest the potential for this type of strategy in control of spoilage fungi. There may be promise in some applications for finding synergies among existing, chemically-related preservatives: work with weak acids has revealed that some work predominantly by acidification, others by targeting respiration, *i.e.*, different primary modes of action against spoilage yeasts (Stratford *et al.*, 2020).

#### 4.2.3 Supply-chain risk assessment

Risk assessment through the supply chain can also help in the preservation of stored foods. Quantitative microbial risk assessments (QMRAs) are becoming common practice in the food industry, encompassing the use of mathematical modelling to predict and help intercept spoilage by moulds (Dagnas and Membré, 2013; Gougouli and Koutsoumanis, 2017) and yeasts (Buehler *et al.*, 2018; Mertens *et al.*, 2012). Specifically, a supply-chain risk assessment would require knowledge on the prevalence of fungi (*e.g.* in raw materials and production environments) as well as knowledge regarding the prevalence of preservation resistance in fungal populations, encompassing biodiversity and heteroresistance (Gougouli and Koutsoumanis, 2012; van den Brule *et al.*, 2020; Zwietering, 2015). The rapid and accurate gathering of genome-sequence, transcriptomic and proteomic data has provided important resources to help support this in recent years. However, there are multiple challenges involved with integrating these data into established QMRAs (Haddad *et al.*, 2018). More research is needed on data collection and successful integration of QMRAs to guide and inform the appropriate management strategies (*e.g.* timing of preservative application during processing) to best protect food from fungal spoilage.

## 5 Concluding perspectives

Fungi pose a serious threat to ensuring food security for a growing global population. Impacts of climate change exacerbate challenges in crop management and food storage, potentially associated with emerging disease- and spoilage-causing strains. Additionally, regulations for chemical treatments are tightening to limit the accumulation of fungicides and of disinfection by-products in food produce, in irrigation, processing water and in the environment. New technologies for fungal control are also necessitated by the emergence of resistance. The development of natural product fungal inhibitors holds promise, helping to address both consumer and regulatory concerns while potentially revealing novel biological activities of unpredicted chemistries. More research is needed to identify and develop candidate molecules. Existing fungal treatments can be improved by exploiting synergies between chemical agents, allowing lower chemicals usage. Identifying novel synergies and commercialising existing synergistic combinations should strengthen current crop management strategies. Concerns over chlorination by-products from conventional chlorine treatments in food processing can be circumvented with electrolysis-based technologies. Development of sanitiser technologies with reduced FAC load while ensuring sufficient stability and safety is crucial, and monitoring of by-product formation is necessary. Technological advances in the delivery of physical decontamination methods could offer alternative or ancillary methods of fungal control. Biocontrol and methods of stimulating plant defence can help reduce both food spoilage and crop disease, while films and coatings may protect fruit from mechanical damage and can be adapted for improved delivery of other control measures. Passive anti-attachment materials offer an exciting possibility for reducing the risks associated with resistance development and deployment of bioactive chemicals, and there is good scope for further refinement of suitable chemistries and material properties. The challenges presented in this focused review emphasise the importance of good management practices in the food industry that minimise contamination, particularly from contaminated water sources, manure usage and worker hygiene (FDA, 1998). Reducing the initial fungal load, in combination with some of the newer strategies outlined here, can contribute to ensuring food safety and supply and reducing the chemical and microbiological loads in already scarce water resources. Such strategies are therefore important steps towards future-proofing the food supply chain from the evolving threats posed by fungi.

## Declaration of competing interest

The authors declare that they have no known competing financial interests or personal relationships that could have appeared to influence the work reported in this paper.
